# From fecal microbiota transplantation toward next-generation beneficial microbes: The case of *Anaerobutyricum soehngenii*

**DOI:** 10.3389/fmed.2022.1077275

**Published:** 2022-12-05

**Authors:** Koen Wortelboer, Annefleur M. Koopen, Hilde Herrema, Willem M. de Vos, Max Nieuwdorp, E. Marleen Kemper

**Affiliations:** ^1^Department of Experimental Vascular Medicine, Amsterdam UMC, Location AMC, University of Amsterdam, Amsterdam, Netherlands; ^2^Amsterdam Cardiovascular Sciences, Diabetes and Metabolism, Amsterdam, Netherlands; ^3^Amsterdam Gastroenterology Endocrinology Metabolism, Endocrinology, Metabolism and Nutrition, Amsterdam, Netherlands; ^4^Department of Pharmacy, Amsterdam UMC, Location AMC, University of Amsterdam, Amsterdam, Netherlands; ^5^Department of Vascular Medicine, Amsterdam UMC, Location AMC, University of Amsterdam, Amsterdam, Netherlands; ^6^Laboratory of Microbiology, Wageningen University, Wageningen, Netherlands; ^7^Human Microbiome Research Program, Faculty of Medicine, University of Helsinki, Helsinki, Finland; ^8^Diabetes Center, Department of Internal Medicine, Amsterdam UMC, Location VUMC, Vrije Universiteit Amsterdam, Amsterdam, Netherlands

**Keywords:** *Anaerobutyricum soehngenii*, *Eubacterium hallii*, next-generation probiotic, live biotherapeutic product, fecal microbiota transplantation

## Abstract

The commensal gut microbiota is important for human health and well-being whereas deviations of the gut microbiota have been associated with a multitude of diseases. Restoration of a balanced and diverse microbiota by fecal microbiota transplantation (FMT) has emerged as a potential treatment strategy and promising tool to study causality of the microbiota in disease pathogenesis. However, FMT comes with logistical challenges and potential safety risks, such as the transfer of pathogenic microorganisms, undesired phenotypes or an increased risk of developing disease later in life. Therefore, a more controlled, personalized mixture of cultured beneficial microbes might prove a better alternative. Most of these beneficial microbes will be endogenous commensals to the host without a long history of safe and beneficial use and are therefore commonly referred to as next-generation probiotics (NGP) or live biotherapeutic products (LBP). Following a previous FMT study within our group, the commensal butyrate producer *Anaerobutyricum* spp. (previously named *Eubacterium hallii)* was found to be associated with improved insulin-sensitivity in subjects with the metabolic syndrome. After the preclinical testing with *Anaerobutyricum soehngenii* in mice models was completed, the strain was produced under controlled conditions and several clinical studies evaluating its safety and efficacy in humans were performed. Here, we describe and reflect on the development of *A. soehngenii* for clinical use, providing practical guidance for the development and testing of NGPs and reflecting on the current regulatory framework.

## Introduction

The commensal gut microbiota play an important role in human health and well-being, regulating host metabolism, shaping our immune system and preventing pathogen colonization (1–3). However, disruption of the intestinal microbiota has been implicated in several diseases, such as gastrointestinal disorders, metabolic disorders and even autoimmune diseases (4, 5). Over the past decades, fecal microbiota transplantation (FMT) has emerged as a potential treatment strategy for such disorders by restoring a balanced and diverse microbiota (6). In addition, FMT has enabled researchers to study causality of the gut microbiota in disease pathogenesis (7, 8). Even though FMT has shown promising results in several diseases (9), the therapy is currently only indicated for the treatment of recurrent *Clostridioides difficile* infections (10). Furthermore, FMT faces several logistical challenges such as donor screening and (anaerobic) sample processing and storage (11, 12). In addition, there are potential safety risks with FMT, such as the potential transfer of pathogenic microorganisms missed during donor screening (13). Other potential risks include the potential transfer of unwanted phenotypes such as obesity or an increased risk of developing disease later in life such a colorectal cancer (14–16).

Due to these limitations and risks of FMT, a more controlled, personalized mixture of beneficial microbes might prove a better alternative. Traditional probiotics are believed to be beneficial for the host health by supporting a balanced microbiota, contributing to the health of the digestive tract and immune system and counteracting pathogenic bacteria through various mechanisms (17–19). However, even though decades of extensive studies have led to numerous prophylactic and therapeutic health claims (20, 21), clinical trials of high methodological quality report conflicting results and debatable conclusions (22). In addition, the majority of the probiotics currently sold on the market contain microorganisms from the *Lactobacillus* and *Bifidobacterium* genera, while these genera constitute only a minor proportion of the human intestinal microbiota (23, 24).

With increasing knowledge of the gut microbiota through affordable genome and metagenome sequencing and the development of better culturing techniques, the list of endogenous microbes with potential health benefits has dramatically increased. Since these microbes are endogenous to the host, they are more likely to engraft and be metabolically active. Even though most of these commensal microbes are still at an early stage of mechanistic investigation, there have been several reports of beneficial microbes restoring the balance of the intestinal ecosystem and improving disease phenotype (25–30). These microorganisms without a long history of safe and beneficial use are commonly referred to as next-generation probiotics (NGP) or live biotherapeutic products (LBP) (31).

Previously, our group performed a randomized controlled trial studying the effects of lean donor FMT in human obese, insulin resistant subjects (32). In line with an improved insulin sensitivity, we observed an increased abundance of the commensal *Anaerobutyricum* spp. [previously named *Eubacterium hallii* (33)] in the small intestine upon allogenic FMT compared to autologous FMT. We thus set out to further study and develop this potential beneficial microbe and focused on *Anaerobutyricum soehngenii* L2-7 among others since it was best characterized (34–36). After confirming a dose-dependent improvement of insulin sensitivity and safety of *A. soehngenii* in a mouse model (37), the strain was produced under controlled conditions and tested in a dose-escalating phase I/II clinical trial (38). Here, we describe the development of *A. soehngenii*, from the identification and production to the first clinicals trial in humans. In addition, we provide a practical roadmap for the development and testing of similar NGPs and reflect on the current regulatory framework.

## Definition of next-generation probiotics and live biotherapeutic products

**FIGURE 1:**
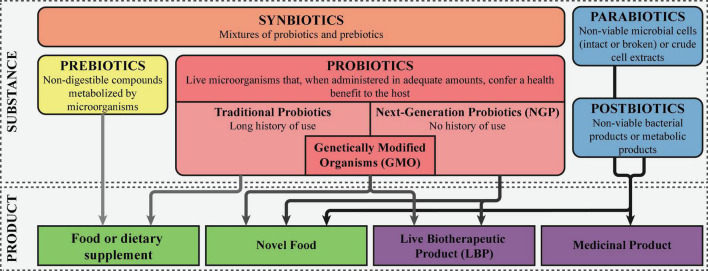
Definitions of probiotics, next-generation probiotics, and live biotherapeutic products. The different “biotics” are colored orange, here denoted as the active substance. The final products are colored green, with the darker green corresponding with products that are considered drugs, while the lighter green falls within the food and food supplements regulation.

The traditional probiotics are defined as “live microorganisms that, when administered in adequate amounts, confer a health benefit on the host”(39). These microbes have a long history of use and are regarded as safe, having a Generally Regarded as Safe (GRAS) status in the United States or a Qualified Presumption of Safety (QPS) status in the European Union (40). In contrast, NGPs are microorganisms without a long history of safe and beneficial use, that like traditional probiotics, confer a health benefit on the host when administered in adequate amounts (31). In 2012 the United States Food and Drug Administration (FDA) introduced the term live biotherapeutic products (LBP), defined as “a biological product that: (1) contains live organisms, such as bacteria; (2) is applicable to the prevention, treatment, or cure of disease or condition of human beings; and (3) is not a vaccine” (41). This FDA guidance statement was followed up in the European Union in 2019, where LBPs were defined as “medicinal products containing live micro-organisms (bacteria or yeasts) for human use” in the European Pharmacopeia (Ph. Eur.) (42). However, since LBPs comprise besides the microorganism also the formulation of the final product and are defined as a medicinal product, this term should not be systematically used to replace NGPs. The term NGP is more extensive, including both the microorganisms present in LBPs and those currently being investigated, not formulated in a final product yet (31). In addition, NGPs could be employed both as a food supplement like traditional probiotics or as a medicinal product in the prevention, treatment, or cure of disease. Finally, genetically modified microorganisms can be viewed a NGPs as well, although the route to market as an LBP is most likely. Figure 1 schematically depicts the various definitions.

## Discovery and isolation of *Anaerobutyricum soehngenii*

In line with the worsening global obesity pandemic, the incidence of the metabolic syndrome has dramatically increased, predisposing individuals to developing cardiovascular diseases and type 2 diabetes (43). Dybiosis of the gut microbiota, defined as a perturbation of the composition and function, has been associated with the emergence of metabolic syndrome (44–46). To further investigate a causal role of the gut microbiota in metabolic syndrome, we previously infused fecal microbiota from lean healthy donors to male subjects with metabolic syndrome (32). Six weeks after the infusion of donor microbiota, peripheral insulin sensitivity increased along with levels of butyrate-producing bacteria, as compared to the autologous FMT group. Among these butyrate-producing bacteria, *Anaerobutyricum* spp. were more abundant in the small intestine, pointing toward a potential role in regulating insulin sensitivity through butyrate production. Since insulin resistant metabolic syndrome subjects are characterized by reduced levels of short-chain fatty acid (SCFA)-producing bacteria (47, 48) and oral supplementation with butyrate improved insulin resistance and dyslipidemia in diet-induced obese mice (49, 50), we concluded that *A. soehngenii* could be a promising NGP to improve insulin-resistance.

Isolated from the feces of an infant in 1996 (34), *A. soehngenii* strain L2-7, previously designated *E. hallii*, is a strict anaerobic, Gram-positive, catalase negative bacterium within the family *Lachnospiraceae* (33). *A. soehngenii* is part of the core microbiota of the human gastrointestinal tract (51, 52). In contrast to other well-known butyrate-producing species such as *Roseburia* and *Faecalibacterium* spp. that produce butyrate from sugars, *A. soehngenii* has the capacity to utilize D- and L-lactate in the presence of acetate instead (53). In addition, the genome contains bile acid sodium symporter and choloylglycine hydrolase genes, suggesting that *A. soehngenii* can affect host bile acid metabolism (54).

The *A. soehngenii* strain (previously *E. hallii* L2-7^T^) was obtained from collaborators in the UK (34, 55) and is available from the DSMZ (Deutsche Sammlung van Mikroorganismen und Zellkulturen) as DSM 17630. The strain was cultured routinely under anaerobic conditions using a previously published protocol (33). Next, we thoroughly characterized the strain. First, the complete genome was sequenced (54), leading to a better understanding of the genetic potential underlying its metabolic capabilities. Next, optimum growth temperature and pH were determined, as well as the tolerability to oxygen. Cell morphology, motility and spore formation were studied using an (electron) microscope and the resistance to heat inactivation and antibiotic susceptibility were determined. Fermentation end products on various carbohydrates were measured and the resistance to bile acids was determined. Finally, the cellular fatty acid contents and the type of peptidoglycan membrane were determined. The results of this thorough characterization led to the reclassification of the previously designated *E. hallii* type strain L2-7^T^ to *A. soehngenii* type strain L2-7^T^ (33).

The metabolic features of *A. soehngenii* were further characterized by proteomic profiling, revealing the complete pathway of butyrate production from sucrose, sorbitol and lactate (56). This analysis identified a new gene cluster, *lctABCDEF*, which was induced upon growth on D, L-lactate plus acetate. Comparative genomics showed this gene cluster to be highly conserved in only *Anaerobutyricum* and *Anaerostipes* spp., suggesting *A. soehngenii* is adapted to a lifestyle of lactate plus acetate utilization in the human gastrointestinal tract (56). The capability to convert potentially harmful D- and L-lactate (57, 58) to the beneficial SCFA butyrate (59) confirmed that *A. soehngenii* was a promising NGP for further preclinical development.

### Learning points and directions

There are two strategies commonly being employed for the development of NGPs. The first method is to associate the presence of a specific strain with a health phenotype and explore whether that strain has a causal effect on the disease phenotype. To date, many NGP candidates have been identified using sequencing technologies to select strains with a depleted abundance in diseased subjects or strains that are associated with successful FMT treatment (60). The second strategy is to adopt a well-characterized probiotic strain and genetically modify the strain to confer a health benefit, e.g., through production and delivery of bioactive molecules (23). The latter approach will lead to a genetically modified organism (GMO) that is subject to specific regulations in various parts of the world, such as in the EU (61–63).

**FIGURE 2:**
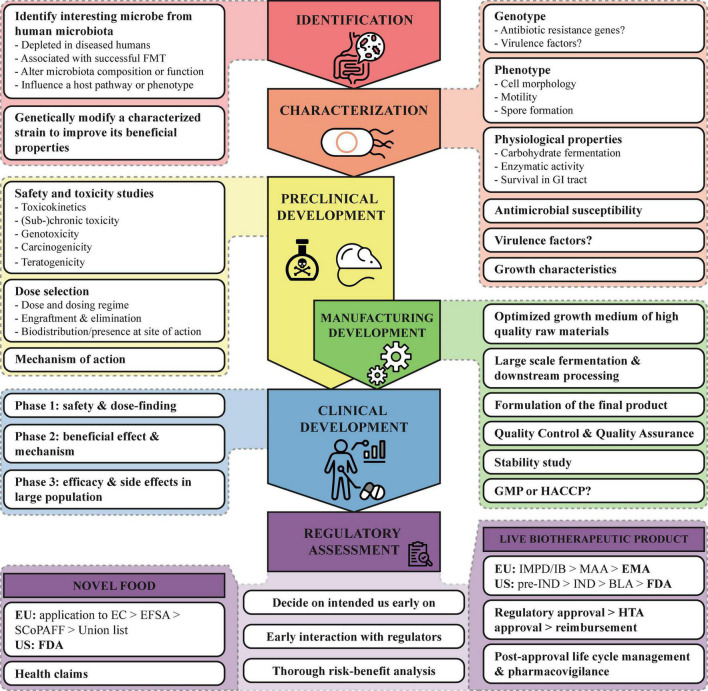
Roadmap for the development of NGP. Important points to consider for the development of NGPs are summarized from the identification to the regulatory assessment. BLA, Biologics License Application; EC, European Commission; EFSA, European Food Safety Authority; EMA, European Medicines Agency; EU, European Union; FDA, Food and Drug Administration; GI, gastrointestinal; GMP, Good Manufacturing Practices; HACCP, Hazard Analysis and Critical Control Points; HTA, Health Technology Assessment; IB, Investigators Brochure; IMPD, Investigational Medicinal Product Dossier; IND, Investigational New Drug; SCoPAFF, Standing Committee on Plants, Animals, Food and Feed, and US, United States.

Regardless of the strategy used to identify or generate the NGP, before any health benefits can be studied *in vivo* the candidate strains need to be fully characterized *in vitro* (64). Figure 2 summarizes the most important characteristics which have to be assessed besides genotyping and phenotyping the strain. In addition, the strain origin and subsequent manipulation or genetic modifications have to be documented. If there are any antimicrobial resistance genes or virulence genes present, the potential for transmission to other microorganisms of the human microbiota should be assessed, as well as measures taken to mitigate this risk. When the NGP is intended to be used in diseased persons with e.g., epithelial barrier damage of immunosuppression, the risk for bacterial translocation should be determined. A thorough strain characterization is critical for the assessment of the potential safety issues concerning the use of the NGP in healthy or diseased humans.

## Preclinical development of *Anaerobutyricum soehngenii*

After *in vitro* testing of *A. soehngenii*, we moved to an animal model to assess safety and efficacy of the strain on insulin sensitivity. First, we manufactured a preclinical batch of *A. soehngenii* under anaerobic conditions as previously described (33). In short, cultures were grown under anaerobic conditions to the end of the exponential phase, concentrated by anaerobic centrifugation, washed with phosphate-buffered saline (PBS) and finally diluted in 10% glycerol to concentrations of 10^6^, 10^8^ and 10^10^ colony-forming units (CFU) in 100 μl. Purity was assessed by 16S rRNA sequencing and microscopic evaluation of cellular morphology. Viability was assessed by most probable number (MPN) analysis and confirmed by microscopic analysis. Samples were directly stored at −80°C and used within 6 months of production, during which time viability was stable. In addition, some of these samples were tested for stability during 2 years to support the product development for the clinical trial.

Next, we performed a dose-finding study in male diabetic (db/db) mice to test the safety and efficacy of orally administered *A. soehngenii* on insulin sensitivity and lipid metabolism (37). Mice were treated daily with *A. soehngenii* or placebo (10% glycerol) for up to 4 weeks, during which time no adverse events were observed (normal vital signs). A significant improvement on insulin sensitivity was observed during the insulin tolerance test, which was strongest for the 10^8^ CFU dose. This was accompanied by a decrease in hepatic fat and a reduced expression of the *Fasn* and *Acc1* genes, both involved in lipogenesis.

To confirm these findings and further dissect the therapeutic mechanism of *A. soehngenii*, a second study with db/db mice was performed independently by the lab of prof. Bäckhed (Gothenburg) (37). Mice were treated with either 10^8^ CFU of *A. soehngenii* or heat-inactivated *A. soehngenii* for 4 weeks. An increase in resting energy expenditure was observed after active *A. soehngenii* treatment, while bodyweight remained identical. In addition, active *A. soehngenii* increased fecal butyrate levels and modified bile acid metabolism as compared to the heat-inactivated *A. soehngenii*. These two mouse studies have shown that treatment with *A. soehngenii* is safe and exerts beneficial effects on metabolism, potentially mediated by butyrate production and changes in bile acid metabolism. These data were used to obtain ethical approval for the clinical studies that we performed in humans.

More recently, a toxicological safety evaluation for *A. soehngenii* CH106, a tetracycline-sensitive derivative from *A. soehngenii* type strain L2-7^T^, has been performed to show that the intake at the recommended dosages is safe (65). As required by the European Food Safety Authority (EFSA) and FDA for safety assessment of new nonabsorbable food ingredients, *A. soehngenii* was assessed for genotoxic potential and subchronic toxicity (66, 67). Both the bacterial reverse mutation and *in vitro* mammalian cell micronucleus tests showed no genotoxic effects. Furthermore, the 90-day subchronic toxicity in rats did not find any adverse events related to the feeding with *A. soehngenii*, not even at the highest dose (5 × 10^11^ CFU/kg body weight/day) exceeding human recommended daily intake more than 100-fold (65). These findings support that oral intake of *A. soehngenii* as food supplement is safe.

### Learning points and directions

During the preclinical development, adequate information on pharmacological and toxicological properties should be generated to support the proposed clinical trial(s). However, safety and toxicity studies with NGPs are challenging. Since the product generally does not reach the systemic circulation, but its metabolites or its activity could directly or indirectly influence physiological functions in the body, efficacy and toxicity are not necessarily related to the dosage. In addition, other factors such as the human physiology and microbiota composition might influence the safety and efficacy. Furthermore, since most NGPs have coevolved with the human host, the holobiont concept, it is difficult to translate the results from animal studies to the human setting (68–70). Therefore, it is highly recommended to combine *in vitro*, *ex vivo* and *in vivo* models to establish a global safety profile adapted to the risks within the intended population. It is common to perform the safety and toxicity studies according to the Organization for Economic Co-operation and Development (OECD) principles for Good Laboratory Practice (GLP). However, due to the need for innovative methods and models (e.g., an artificial model of the human gastrointestinal tract) which may not be validated nor at GLP level, this might prove difficult (71).

For food ingredients and dietary supplements, the EFSA advices a tiered approach for toxicological studies (67). This tiered approach evaluates the toxicokinetics, genotoxicity, subchronic and chronic toxicity, carcinogenicity and teratogenicity of the NGP, balancing data requirements against the risk. This approach was used as well for the toxicological safety evaluation for *A. soehngenii* CH106 (65). If the NGP is intended to be used as medicinal product in a diseased population, it is important that safety for the targeted population is demonstrated. Figure 2 summarizes the most important issues that have to be addressed, such as the effect of dosage and duration of treatment on toxic response and the teratogenic, carcinogenic and genotoxic potential.

## Manufacture of *Anaerobutyricum soehngenii* suitable for clinical testing

Before we could orally administer *A. soehngenii* to humans, a product suitable for a clinical trial had to be manufactured. At the time of approval by the independent ethics committee (2014), A. soehngenii was regarded as a probiotic and had to comply with the Dutch “Warenwet” (72), which was in line with the EU regulations for dietary supplements (73). This meant the manufacturing had to be performed according to Hazard Analysis and Critical Control Point (HACCP) standards (74). Therefore, we contracted a third-party manufacturer, which was ISO 9001 accredited and had ample experience with the fermentation of probiotic strains for clinical intervention studies under HACCP standards.

### Growth medium

First of all, the growth medium was further optimized for large scale production of a food-grade product. The composition was based on previous experience (33), whereby (1) laboratory chemicals were converted to food-grade sources, (2) only animal-free components were used (no heme or meat peptone), (3) complexity was reduced (removal/reduction of trace minerals, vitamins, carbon sources and organic acids) and (4) the biomass yield was further improved. Raw materials were sourced from audited, reliable suppliers to ensure high quality. Before fermentation, the growth medium was prepared and sterilized inside a large fermenter system, which was made completely anaerobic by nitrogen (N2) flush.

### Fermentation

**FIGURE 3:**
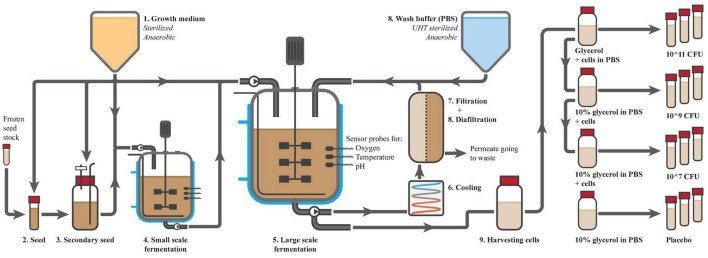
Preparation of Anaerobutyricum soehngenii. Production started with the production of a sterile anoxic growth medium (1), which was used for the seeding (2 and 3) and fermentations (4 and 5) of *A. soehngenii*. The fermentations were well controlled for temperature, presence of oxygen and pH amongst others. After the final fermentation, cells were cooled (6), concentrated (7) and washed by diafiltration (8). Cells were harvested from the fermenter (9), diluted with glycerol (10% final concentration) to 10^10^ CFU/ml and dispensed over 10 ml vials that were labeled and directly frozen. The highest concentration was used to make dilutions for the lower doses.

Fermentation was performed in four sequential steps, which are depicted in Figure 3. First, a small volume of food-grade medium was inoculated with a carefully prepared frozen seed stock of *A. soehngenii*. The same strain was used in the animal studies and had therefore been well characterized, was viable, pure and free of any bacterial of viral contaminants. After 24 h of fermentation at 37°C, the culture was used to inoculate 1 L of medium, which was again fermented for another 18 h. Then, this secondary seed culture was used to inoculate 30 L of medium in a small fermenter, which was fermented for 17 h and which acted as a test run for the large-scale fermentation. Finally, 290 L of medium in the large fermenter was inoculated with 10 L of inoculum of the small fermenter. Both small and large fermenters were controlled for temperature, pH and oxygen level and the optical density (OD) of the culture was used to determine the fermentation time (between 14 and 18 h). After 16 h of fermentation in the large fermenter, *A. soehngenii* grew to an OD of approximately 10.

### Concentration and washing

Using hollow fiber membranes (Koch membrane systems; HF3043-25-43-PM500; HF3043-16-106-PM500) and diafiltration with PBS, the cells were concentrated and washed. The fermentate was cooled to 10°C, pumped through the anaerobic membrane unit and concentrated to 40–50 L within 3 h. During the second phase diafiltration was performed to reduce the levels of medium components and fermentation products. Wash buffer was sterilized using ultra-high temperature, de-aerated and directly added to the returning cell flow into the fermenter. After 6 h, the cells were concentrated about 20-fold to 15 L and 99.8% of medium compounds were discarded to waste, leaving solely 2.9% of medium components in the final concentrate. Finally, 9 L of product could be harvested from the system into a sterile, N2-flushed container of 10 L.

### Preparation of end-product

Four different batches were produced for the clinical study, consisting of 600 tubes with 10 mL *A. soehngenii* in concentrations of 10^6^, 10^8^, and 10^10^ CFU/ml in PBS + 10% glycerol and one placebo batch with only 10% glycerol in PBS. For every batch 7 L bottles were prepared with glycerol and PBS for further dilution, which were autoclaved, cooled and flushed with N2. From the 9 L harvested concentrate, the necessary volume was added to these bottles to obtain the correct concentration. Bottles were placed on ice, under continuous stirring and N2 flush. The 10 mL tubes were first filled with N2, followed by 10 mL of product using a dosing-tube-pump. Tubes were immediately closed, labeled and placed in a freezer at −30°C within 10 min of filling. All filling was performed inside a disinfected laminar flow cabinet.

### Quality control

During the manufacturing, there was a continuous monitoring of temperature, pH and oxygen level. In addition, the cell count and OD were determined at every step during the process, as well as the absence of any contaminants. Since anaerobes are hard to enumerate quantitatively on agar plates, an MPN analysis was performed under anaerobic conditions to obtain the number of viable cells and cell morphology was assessed microscopically. All above quality controls were performed for the packaged vials, which complied with the standards for human consumption. Table 1 shows the specifications that were defined for the intermediates and final product.

**TABLE 1:** Specifications for the *Anaerobutyricum soehngenii* intermediates and final product.

Test	Method	Acceptance criteria	Intermediate (I), product (P), or stability (S)
Identity	Genome sequencing	Confirm strain is *A. soehngenii L2-7*	I*
	Microscopy (visual observation)	Complies with phenotypic characteristics *A. soehngenii L2-7*	I, P
Potency	Culturing/MPN	10^10 CFU/ml	P, S
Purity	Microbial contamination	*Salmonella* spp.: absent *Listeria monocytogenes*: absent *Enterobacteriaceae*: <10 CFU/ml Coagulase-negative *Staphylococci*: <10 CFU/ml *Bacillus cereus*: <10 CFU/ml	I, P, S
Other	pH	6.0–7.0	I, P
	Storage	Vial with 10 ml suspension, stored at −20°C	P
	Labeling	According to GMP annex 13	P

*The complete genome of the strain used for seeding has been completely sequenced. CFU, colony-forming unit; GMP, good manufacturing practice.

Subsequently, the stability of the produced vials was tested every 6 months. After production, the vials were given a “best before” date of 6 months, which is required by law for food products in the Netherlands. This gave us the opportunity to extend the expiration date of the vials if the viability and purity criteria were met. Table 2 shows the potency and purity of the vials with the highest dose *A. soehngenii* during a 3-year time period.

**TABLE 2:** Results of stability testing (potency and purity) of *A. soehngenii*.

Storage time (months)		6	12	18	24	30	36
Potency	MPN (CFU/ml)	1.0E+09	1.0E+09	1.0E+09	1.0E+10	1.0E+09	1.0E+09
	Microscopy	Normal	Normal	Normal	Normal	Normal	Normal
Purity	*Salmonella* spp.	Absent	Absent	Absent	Absent	Absent	Absent
	*Listeria monocytogenes*	Absent	Absent	Absent	Absent	Absent	Absent
	*Enterobacteriaceae* (CFU/ml)	<10	<10	<10	<10	<10	<10
	Coagulase-negative *Staphylococci* (CFU/ml)	<10	<10	<10	<10	<10	<10
	*Bacillus cereus* (CFU/ml)	<10	<10	<10	<10	<10	<10

MPN, most probable number; CFU, colony-forming unit.

### Learning points and directions

Producing a strain at industrial scale sets different requirements for strains and culture media than laboratory scale culturing (75). Therefore, when a strain qualifies as potential NGP, steps should be taken to see if the strain can be cultured at an industrial scale. The strict conditions necessary for culturing NGPs are one of the technical challenges, such as the need for specific nutrition, the absence of oxygen, a stable temperature and a suitable pH (24). In addition, longer hold times, sheer stress from pumping, the downstream purification processes and storage may negatively impact the viability of the bacterial cells. Next, the strains have to be incorporated into a product, such as capsules, a powder or liquid suspension. Since most NGPs are strict anaerobes or facultative anaerobes, the exposure to oxygen should be kept to a minimum. To this end, oxygen permeability into containers should be reduced and antioxidants could be added to reduce the redox potential (76). Upon ingestion of the product, NGPs have to survive the harsh environment of the gastrointestinal tract. Enteric-coated capsules and microencapsulation are useful strategies to protect the bacteria and deliver them to their site of action (77, 78). Ultimately, manufacturing needs to result in a robust and stable product that will allow for delivery of the NGP in sufficient numbers for an efficacious dose until the expiration date (75).

For medicinal products or LBPs, production according to Good Manufacturing Practices (GMP) is required (41). For foods and dietary supplements, production in HACCP-certified plants is the standard (74). Regardless, quality control and quality assurance programs needs to be in place to ensure a consistent quality of ingredients and final product and to secure a reliable production process (75). The manufacturing process of the strain should be clearly documented, from the raw materials used, the cell bank system, growth and harvesting of the cells, purification and downstream processing to the in-process testing. Likewise, the manufacturing of the final product has to be thoroughly described, including production records and instructions for formulation, filling, labeling and packaging. For both the strain and product manufacturing, the risks for cross-contamination with other products produced in the same rooms or with the same contact equipment has to be assessed. Specifications for the strain and product have to be described, including a description of sampling procedures and the validated test methods. These specifications should describe the identity, potency, purity, contamination, appearance and, if applicable, additional tests for percentage of viable cells, particulate matter, pyrogens, pH and residual moisture. Furthermore, stability data has to be generated, demonstrating the product is stable for the planned duration of use with regards to potency and contamination. For frozen products, the influence of multiple freeze-thaw cycles should be assessed, while for lyophilized products the shelf life after reconstitution should be explored. Finally, the impact of the product on the environment needs to be assessed, especially when the strain is genetically modified, pathogenic, ecologically more fit than the wildtype, or difficult to eradicate.

## Clinical trials with *Anaerobutyricum soehngenii*

### Safety/dose-finding trial

To validate the murine data in a human setting, we set up a single-blinded, phase I/II dose-escalation trial to determine safety and efficacy of *A. soehngenii* in obese, insulin-resistant subjects (38). In this study, 27 obese Caucasian males with the metabolic syndrome were included and assigned to receive *A. soehngenii* in increasing dose of 10^7^, 10^9^, or 10^11^ cells/day for 28 days. While subjects were blinded for their respective treatment dose, first 9 subjects had to successfully complete the study protocol on the lowest dose before the dose was escalated to a higher concentration. Subjects stored the frozen vials with *A. soehngenii* at −20°C at home and every day a single 10 mL vial was thawed, mixed with 100 mL of milk and consumed orally. The milk was added to increase the pH in the stomach and thereby protect the living cells during gastrointestinal passage (79). The primary outcome was safety and in addition the impact on insulin sensitivity and lipolysis was assessed after 4 weeks of treatment.

Treatment with *A. soehngenii* up to 10^11^ cells/day was well tolerated without any serious adverse events (38). When all treatment groups were combined, the fecal abundance of *A. soehngenii* correlated with an improved peripheral insulin sensitivity, accompanied by beneficial changes in the bile acid profile. Unexpectedly, no increase in fecal butyrate levels was observed, which could be explained by the volatility of SCFAs and the assays’ detection limits making butyrate difficult to measure. The increase in (fecal) *A. soehngenii* abundance was transient and mostly gone 2 weeks after cessation. The viability of the administered strain was negatively affected by stomach acid and oxygen. However, *A. soehngenii* was partially able to survive the gastrointestinal passage as indicated by the highest replication signal in the feces of subjects that received the highest dose. The viability (and therapeutic efficacy) could be further improved by protecting the strain better from the acidic and oxygenic environment through encapsulation and/or freeze-drying.

### Different administration method and mode of action

To further elucidate the mode of action of *A. soehngenii* in humans, a randomized placebo-controlled crossover trial was performed in which the strain was directly administered in the duodenum, thereby circumventing the stomach acid and reducing the exposure to oxygen (80). Since the small intestine plays a central role in glucosensing, regulation of insulin sensitivity/secretion and glucose homeostasis, it was hypothesized that a direct duodenal infusion of *A. soehngenii* could further enhance the therapeutic effect (81). Again, obese subjects with the metabolic syndrome (*N* = 12) were included and randomized to a single nasoduodenal infusion with the highest dose of *A. soehngenii* (10^11^ cells) or placebo (10% glycerol in PBS). After 6 h, a duodenal biopsy and mixed meal test was performed. In addition, subject monitored their 24-h glucose and collected several fecal samples. After a 4-week washout period subjects switched to the other treatment arm, which was determined long enough to lose the strain during the first trial.

Again, this study showed that administration of *A. soehngenii* was safe and well-tolerated. Treatment with the strain increased postprandial excursion of insulinotropic hormone glucagon-like peptide 1 (GLP-1), which was accompanied by a reduced glucose variability (80). Given that *A. soehngenii* has the capacity to produce butyrate (51, 53) and fecal levels of butyrate tended to be higher following *A. soehngenii* treatment (80), the increased GLP-1 secretion could be the result of butyrate activating the G protein-coupled receptor 43 (GPR43) on intestinal L cells (82). In addition, since *A. soehngenii* expresses a bile acid sodium symporter and bile acid hydrolases (54) and plasma levels of secondary bile acids were elevated (80), the increased GLP-1 expression could also be the consequence of Takeda G protein- coupled receptor 5 (TGR5) activation by secondary bile acids (83). Moreover, treatment with *A. soehngenii* led to a decreased duodenal expression of the nuclear farnesoid X receptor (FXR) and its target gene *OSTa*, which may also account for an increased GLP-1 availability (84, 85). Finally, the improvement in glucose variability could be explained by the insulin-sensitizing effects of GLP-1 as well as butyrate (49, 86).

Furthermore, *A. soehngenii* altered the duodenal transcription of 73 genes, most prominently inducing the expression of *REG1B* along with *REG1A*, which encode for generating islet-derived protein 1A/B (80). Being strongly expressed within Paneth cells at the base of intestinal crypts, Reg1A and Reg1B are secreted in the lumen and probably act locally, possibly by inducing progenitor or L- cell hyperplasia (80). Moreover, Induction of *REG1B* was found to correlate with both an increased GLP-1 secretion and a reduced glucose variability 24 h after administration of *A. soehngenii* (80). Treatment with a single dose of *A. soehngenii* did not impact the microbiota composition or diversity, as was also seen in the previous studies. In addition, the abundance of fecal *A. soehngenii* was not altered over time, excluding microbiota-mediated carry-over effects at time of crossover (80).

### Learning points and directions

The main objective of the first clinical studies is to establish safety and to define the appropriate dosage range and regimen based on the tolerability of the product (64). This includes the determination of the minimal effective dose or an optimal effective dose range and, if possible, the maximal safe dose. Besides dosing, the focus should be on obtaining safety data to identify common product-associated adverse events. These early clinical studies are commonly performed in healthy volunteers, although inclusion of patients could be more appropriate, for example when the NGP should correct dysbiosis (64). Risk mitigation measures to ensure the safety of study participants should be taken into account, such as sequential enrollment, dose escalation and monitoring by an independent data monitoring committee. Furthermore, it is expedient to monitor for translocation, inflammation and infection and to establish persistence of NGP and its effects after the final administration.

It is important to account for other confounding factors that influence the function or composition of the microbiota, such as age (87, 88), diet (89), lifestyle (90) and environmental factors (91, 92). In this respect, studies with a placebo-controlled cross-over design are very useful as they can limit the influence of such extrinsic and intrinsic confounding factors, thereby allowing for a smaller sample size. Needless to say, blinding is very important and the washout period should be carefully considered. Increasingly, the baseline microbiota composition is incorporated in the screening criteria as well, looking for example for the presence of specific bacterial groups or clustering within specific enterotypes (93). This will lead to more comparable study groups and can optimize the efficacy of the intervention when a specific bacterial group is involved in the mechanism of action.

## Regulatory framework next-generation probiotics

According to the definition of probiotics by the FAO and WHO, probiotics can be classified as both a dietary supplement and a drug, while there is a profound regulatory difference. Similarly, products with NGPs can reach the market as a food, dietary supplement or drug depending on the intended use. In the EU, foods are regulated by the EFSA and drugs by the EMA, while in the US the FDA deals with both categories. When the intended use is related to the prevention, alleviation or cure of disease, the product will be considered a medicinal product or medical device. In contrast, an orally ingested product with claims relating to enhancement of physiological function or reduction of a disease risk factor could be classified as a functional food or food supplement. Furthermore, topically applied products with a purely cosmetic function could be assessed as a cosmetic. To ensure regulatory compliance, it is important to decide on the indented use and consequent regulatory classification prior to preclinical studies and manufacturing (71).

### Functional food or dietary supplement

In the European Union, “food” is defined as “any substance or product, whether processed, partially processed or unprocessed, intended to be, or reasonably expected to be ingested by humans.” Foods and food ingredients are further subdivided into different categories, such as conventional food, food supplements and novel foods, among others. Each of these categories is regulated accordingly, with general requirements and provisions regarding to labeling, presentation and advertising (73, 94). When NGPs are intended for use as food or dietary supplement, they are most likely considered a novel food, since new strains have not been widely consumed within the EU before May 1997 (95). However, if the NGP has been genetically modified, it will be regulated as a genetically modified food (61).

For an NGP to reach the market as a novel food, it needs to be authorized and included in the Union list (95). One of the most important conditions is that the NGP does not pose a risk to human health, which has to be supported by scientific evidence. This consists of a comprehensive risk assessment, combining biological and toxicological studies in the context of anticipated human exposure to evaluate the potential risk to human health (96). In addition, an application should contain detailed descriptions of the NGP, the manufacturing process, the composition of the product, analytical methods used, labeling and conditions for intended use (95).

Many safety-related aspects have been shown to be common at the species level, which has led to the QPS list of the EFSA, expressing a species-based safety evaluation for microbes used as food (40). If the NGP as a species can be unambiguously identified to a QPS group, the developer does not need to perform detailed tolerance and toxicology studies. However, most NGPs will not belong to a QPS group and must be evaluated by the EFSA to ensure safety (95). Besides safety, the product must not contribute to the spread of antimicrobial resistance in the food chain or environment, requiring phenotypic and genotypic assessment of antimicrobial resistance.

Any health claims for NGPs have to be submitted to a national competent authority and will be passed on to the EFSA for scientific evaluation (97). Even the statement “contains probiotics/prebiotics” is considered a health claim in the EU (93). For a health claim to be accepted, a proper characterization of the NGP is required, as well as a proven beneficial health effect and causal relationship supported by high-quality studies (98).

### Live biotherapeutic product

Since 2012 and 2019 quality requirements for LBPs have been clarified by the FDA and EDQM (41, 42), where LBPs are described as medicinal products containing live microorganisms for human use. Other than these quality requirements, there is currently no specific LBP regulation. However, since LBPs contain live microorganisms, they are considered biological medicinal products and as such have to comply with the legislative and regulatory framework. In absence of a specific LBP subcategory, developers will have to rely on the regulatory concepts available for the other subcategories of biological medicinal products. One of these concepts is a thorough risk-benefit analysis based on quality, safety and efficacy data obtained from preclinical and clinical studies. Cordaillat-Simmons et al. and Rouanet et al. previously elaborated on what a thorough risk-benefit analysis should include (64, 71). Other relevant guidelines for the design of preclinical and clinical studies are the International Council for Harmonization of Technical Requirements for Pharmaceuticals for Human Use (ICH) guideline on general consideration for clinical trials (ICHE8) (99), the Committee for Medicinal products for Human Use (CHMP) guideline on strategies to identify and mitigate risks for first-in-human and early clinical trials with investigational medicinal products (100), and the CHMP guideline on Human Cell-Based Medicinal Products (101).

For an LBP to reach the market in the EU, marketing authorization has to granted through a centralized or a national route. Under the centralized authorization procedure, EMA’s CHMP carries out the scientific assessment, whereafter the European Commission takes a legally binding decision based on EMA’s recommendation. To date, no LBPs have reached the EU market, which is partly due to the lack of a defined regulatory framework. Recently, Paquet et al. published their experiences with both the EMA and FDA leading up to their first-in-human trial (102). They described several key considerations for the development and (non-) clinical testing of LBPs based on points raised by the competent authorities. Furthermore, they highlighted the importance of early interaction with the competent authorities to discuss uncertainties and reduce risks in the absence of clear guidelines.

## Concluding remarks

Above we described our experience with the development of *A. soehngenii* as an NGP and provided several (regulatory) directions. Figure 2 summarizes these points and provides a schematic roadmap for developing NGPs. With the increasing knowledge on our intestinal microbiota, more and more potential NGPs will be discovered and developed, either as novel food/supplement or as LBP. It is important that these new strains are well characterized, of high quality and safe. Though difficult and complex, a thorough safety assessment for NGPs is very important, especially since efficacy and toxicity are not necessarily related to the dosage. Furthermore, since this is a relatively young field and currently no specific LBP regulation, talking to regulators in early stages of development can help to mitigate risks and clarify any uncertainties. This requires a clear view on the route to market (food or drug) early in the development.

We illustrated the development of NGPs with the strict anaerobe *A. soehngenii* as example. Identified as potential beneficial microbe after an FMT intervention, this microbe showed promising results in both preclinical *in vitro* and *in vivo* studies as well as in humans. Treatment with *A. soehngenii* was found to be safe and well tolerated. It showed promising effects on improving insulin sensitivity, increased GLP-1 secretion and reduced glucose variability. These effects are potentially mediated through the production of butyrate and secondary bile acids. By protecting the strain better from the acidic and oxygenic environment, e.g., through lyophilization and encapsulation, the viability and thereby therapeutic efficacy could potentially be increased. This NGP is currently being further developed as a food supplement.

## Author contributions

KW wrote the first draft of the manuscript. All authors contributed to the manuscript revision, read, and approved the submitted version.
